# Therapeutic efficacy of *Nigella sativa* and *Ziziphus lotus*: sustainable strategies for diabetes, antimicrobial resistance, and health treatment

**DOI:** 10.3389/fnut.2025.1592423

**Published:** 2025-09-03

**Authors:** Alaa S. Bedir, Razan S. Almasri, Seham M. Al Raish

**Affiliations:** ^1^Department of Nutrition, College of Medicine and Health Science, United Arab Emirates University, AlAin, United Arab Emirates; ^2^Department of Biology, College of Science, United Arab Emirates University, AlAin, United Arab Emirates

**Keywords:** antimicrobial resistance, diabetes management, medicinal plants, phytochemical analysis, traditional medicine

## Abstract

The pharmacological potential of *Nigella sativa* and *Ziziphus lotus*, two medicinal plants native to the United Arab Emirates (UAE), is explored in the context of sustainable healthcare strategies. Both plants have demonstrated notable antidiabetic and antimicrobial effects in preclinical studies. For instance, thymoquinone from *Nigella sativa* has shown glucose-lowering efficacy comparable to metformin in rodent models, while *Ziziphus lotus* extracts have outperformed acarbose in inhibiting carbohydrate-digesting enzymes. This paper synthesizes findings from ethnobotanical surveys, pharmacological research, and clinical literature to assess their therapeutic relevance. Additionally, it addresses challenges in standardization, sustainable harvesting, and environmental influence on phytochemical composition. While current evidence is promising, gaps remain in clinical validation and regulatory integration. This review aims to inform future research and policy, supporting the incorporation of UAE-native medicinal plants into evidence-based healthcare practices.

## 1 Introduction

Diabetes mellitus and microbial infections remain two of the leading causes of mortality worldwide and pose an escalating public health crisis. According to the International Diabetes Federation (1), approximately 3.4 million deaths were attributable to diabetes in 2024 equating to one death every nine seconds with 43% of adults with diabetes remaining undiagnosed. The World Health Organization reported 1.6 million deaths directly caused by diabetes in 2021, nearly half of which occurred before the age of 70. With ageing populations, sedentary lifestyles, and the global rise in obesity, the number of adults living with diabetes has quadrupled since 1990 and now exceeds 800 million. Global prevalence has doubled from 7 to 14% between 1990 and 2022. This epidemic imposes a substantial economic burden, with global diabetes-related healthcare expenditure surpassing US$ 1 trillion annually and high blood glucose responsible for about 11% of cardiovascular deaths (1, 2).

Microbial infections and antimicrobial resistance (AMR) further intensify the threat. In 2019, infectious diseases accounted for 13.7 million deaths globally, with bacterial infections contributing to 7.7 million of these. Alarmingly, just five pathogens *Staphylococcus aureus (S. aureus)*, *Escherichia coli (E. coli)*, *Streptococcus pneumoniae*, *Klebsiella pneumoniae*, and *Pseudomonas aeruginosa (P. aeruginosa)* were responsible for over half of the mortality (3). Furthermore, 4.95 million deaths were associated with bacterial AMR, with 1.27 million directly attributable to resistant pathogens. Forecasts suggest that without global intervention, multidrug-resistant (MDR) infections may cause up to 10 million deaths annually by 2050, while AMR is projected to contribute at least US$ 1 trillion in additional healthcare costs by mid-century (2, 4).

Patients with type 2 diabetes mellitus (T2DM) are particularly vulnerable to microbial infections due to hyperglycemia-induced immune dysfunction, including reduced neutrophil activity and impaired antioxidant defenses (5, 6). Chronic hyperglycemia promotes tissue damage, poor wound healing, and microbial colonization—conditions that contribute to severe outcomes such as diabetic foot infections (DFIs), urinary tract infections, pneumonia, and sepsis. The clinical management of these infections has become increasingly difficult due to MDR organisms and the formation of biofilms, which enhance microbial persistence and antibiotic tolerance (7, 8).

Current treatment approaches for diabetic infections typically rely on broad-spectrum antibiotics, including beta-lactams, fluoroquinolones, macrolides, and aminoglycosides. In severe or polymicrobial infections, agents such as piperacillin–tazobactam, linezolid, or carbapenems are commonly employed (9, 10). However, increasing resistance and the limited pipeline of new antimicrobials demand urgent exploration of alternative strategies.

Mechanistically, microbial resistance is driven by multiple factors: biofilm matrices limit drug penetration and harbor “persister” cells; efflux systems such as MexAB-OprM in *P. aeruginosa* expel a wide range of antibiotics; and enzymatic degradation via β-lactamases or oxidative stress responses further undermine treatment efficacy (8, 11, 12). Addressing these complex challenges requires innovative and integrated therapeutic solutions.

In response, recent advances highlight the potential of repurposed antidiabetic drugs (e.g., metformin, DPP-4 inhibitors) and plant-derived antimicrobials rich in flavonoids, alkaloids, and polyphenols, which exhibit both hypoglycemic and antibacterial effects (5, 6, 13). Compounds from plants such as *Mangifera indica*, *Prunella vulgaris*, and *Camellia sinensis* have demonstrated antimicrobial action against MDR strains of *E. coli*, *S. aureus*, and *P. aeruginosa* (13). These emerging therapies not only reduce infection risk but also complement existing pharmacological regimens, offering promising alternatives in the fight against diabetes-related infections and the AMR crisis.

Medicinal plants (MP) have historically been pivotal in both traditional and modern medicine, offering a wide array of therapeutic benefits. The rising interest in plant-based treatments reflects the global pursuit of alternative and complementary solutions for addressing chronic health issues, including metabolic disorders, and infectious diseases (14, 15). The United Arab Emirates (UAE), characterized by its unique desert vegetation and rich tradition of herbal medicine, is home to pharmacologically significant plants such as *Nigella sativa (N. sativa)* (black seed) and *Ziziphus lotus (Z. lotus)* (sidr, jujube) (16).

*Nigella sativa*, widely cultivated in the UAE, is renowned for its bioactive compounds such as thymoquinone, quercetin, and p-cymene, which exhibit potent antioxidant, anti-inflammatory, and antidiabetic properties. Similarly, *Z. lotus*, a native species thriving in the region’s harsh climate, contains diverse secondary metabolites, including flavonoids and saponins, known for their antidiabetic and antimicrobial activity (17–20). Notably, *N. sativa* and *Z. lotus* are rich in phytochemicals such as thymoquinone, flavonoids, alkaloids and saponins, which have demonstrated antidiabetic, antimicrobial and antioxidant activities in preclinical studies. These multifunctional properties make them attractive candidates for developing novel botanical therapeutics (14–20). The therapeutic potential of these plants aligns with the pressing need for innovative approaches to mitigate the growing burden of non-communicable diseases and antimicrobial resistance in the UAE (21–23).

This review integrates insights from ethnobotanical studies, pharmacological assays, and clinical research to highlight the antidiabetic and antimicrobial benefits of medicinal plants used in the UAE. By focusing on *N. sativa* and *Z. lotus*, which are deeply rooted in local traditional practices yet scientifically underexplored in a UAE-specific context, this work bridges the gap between ancestral knowledge and contemporary biomedical validation. The review also addresses critical challenges, including the lack of clinical standardization, sustainable harvesting practices, and limited regional data. By doing so, it offers a focused and culturally relevant perspective that contributes to global ethnopharmacological knowledge and supports the integration of native resources into modern therapeutic strategies (17–20).

A major strength of the present review lies in its focus on two medicinal plants, *N. sativa* and *Z. lotus*, which are native to the UAE and have demonstrated promising pharmacological potential in preclinical settings. By integrating ethnobotanical knowledge with scientific evidence, this review highlights the relevance of culturally rooted botanical therapies in addressing global health threats like diabetes and microbial resistance. However, one limitation of the current study is the lack of randomized clinical trials conducted within the UAE, as well as the exclusion of relevant non-English literature. To contextualize the urgency of this topic regionally, recent epidemiological data from a large cross-sectional study showed that nearly 30% of individuals with T2DM in the UAE had established atherosclerotic cardiovascular disease (eASCVD), and over 99% had a high or very high 10-year risk for cardiovascular events (24). These alarming figures underscore the pressing need for sustainable, preventive therapeutic strategies that are locally available, affordable, and culturally acceptable.

To our knowledge, this is the first comprehensive review that integrates pharmacological, phytochemical, and ethnobotanical data on *N. sativa* and *Z. lotus* within the specific cultural and environmental context of the UAE. While both plants have been studied individually in broader regional or global settings, their targeted evaluation in relation to the UAE’s unique healthcare challenges namely the dual burden of diabetes and antimicrobial resistance remains underexplored. This review is novel in its synthesis of UAE-specific data, such as regional prevalence of T2DM and atherosclerotic cardiovascular disease (24), alongside bioactivity evidence drawn from both traditional use and experimental studies. By linking local medicinal resources with global therapeutic needs, this work contributes a culturally relevant, sustainability-focused perspective that bridges traditional healing and modern scientific validation.

## 2 Scientific search methodology

A systematic literature search was conducted to retrieve peer-reviewed studies, reviews, and ethnobotanical reports focusing on the medicinal properties of UAE-native plants specifically *N. sativa* and *Z. lotus*. The search spanned June to December 2024, covering PubMed, Google Scholar, and ScienceDirect databases. Search terms combined the botanical and common names of the plants (e.g., “*Nigella sativa*,” “black seed,” “*Ziziphus lotus*,” “jujube,” “sidr”) with specific keywords such as “antidiabetic,” “antimicrobial,” “traditional medicine UAE,” “bioactive compounds,” “thymoquinone,” and “sustainable therapy.”

Boolean operators (AND, OR) were used to broaden or refine search results. The search strategy also included manual screening of reference lists from relevant articles to identify additional sources. Only English-language publications or those with reliable translations were included to ensure data accuracy, from the last 20 years (2004–2024). Studies included were *In vitro*, *In vivo*, or clinical trials with clear pharmacological, phytochemical, or therapeutic data relevant to diabetes management and antimicrobial activity.

**Figure 1:**
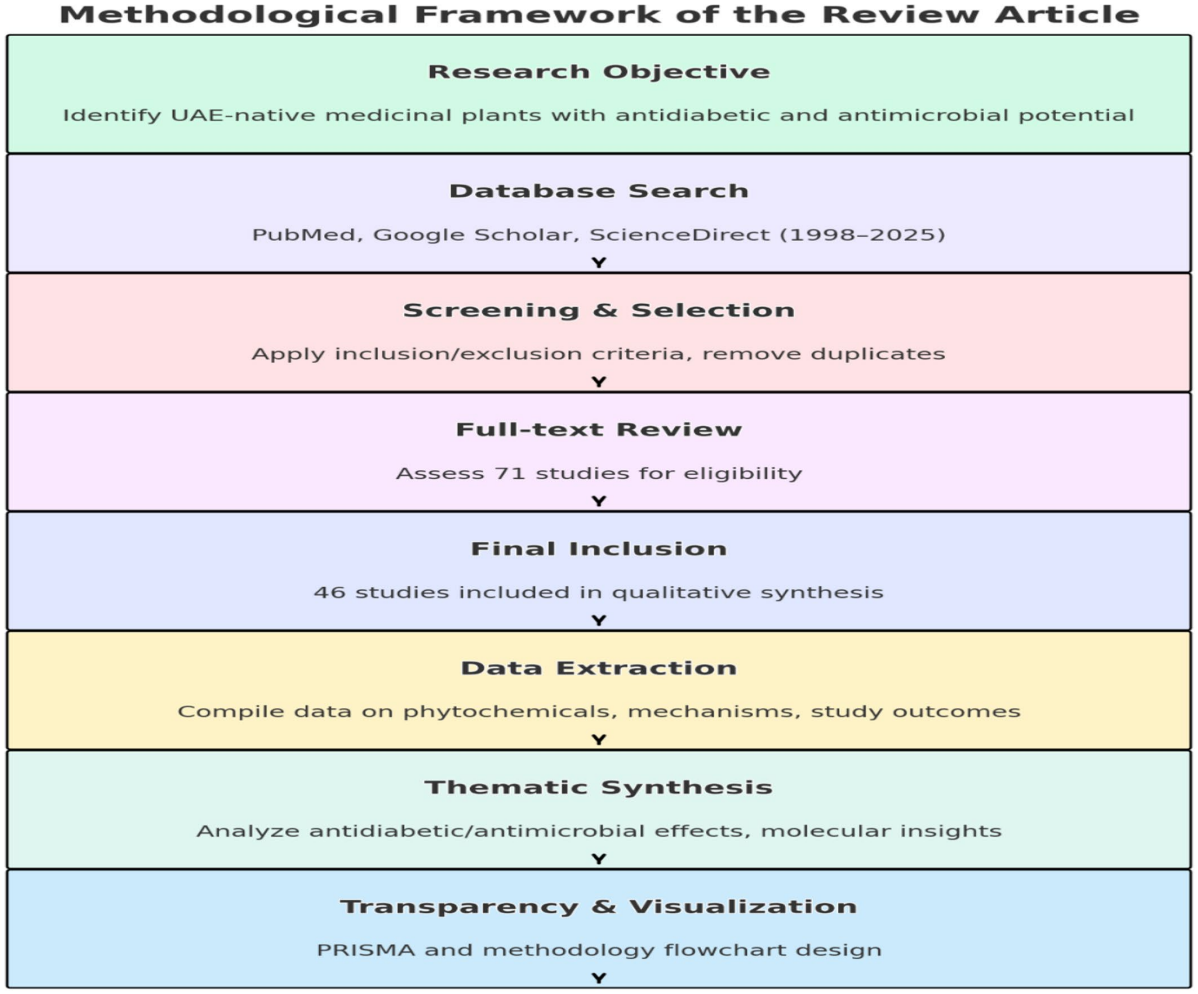
Methodological flowchart summarizing the steps followed in this systematic review, including search strategy, screening, data extraction, and synthesis.

Articles that lacked sufficient data on medicinal effects, focused solely on nutrition or agriculture, or were not related to UAE flora were excluded to ensure regional relevance. A total of 312 studies were initially retrieved. After duplicate removal and eligibility screening based on inclusion/exclusion criteria, 46 articles were included in the final review (see PRISMA diagram, Figure 1, and VOSviewer software, Supplementary Figure 1).

### 2.1 Selection criteria

#### 2.1.1 Data collection

Between June and December 2024, a comprehensive review of the flora of the UAE was conducted, utilizing a diverse range of references, including research and review articles as well as reputable online resources. The initial phase of the review involved compiling an extensive list of botanical names and their synonyms, which served as the foundation for a systematic and detailed literature review. Online platforms such as Google Scholar and databases like PubMed were utilized to identify relevant studies, focusing on plants that are either native to or naturalized within the UAE. The review emphasized the medicinal applications of these plants in both traditional and contemporary contexts. A systematic search strategy was employed, incorporating keywords that linked the botanical names and synonyms of each plant to specific medicinal uses, with particular emphasis on their roles in managing diabetes and combating bacterial infections. This methodical approach ensured a robust and comprehensive exploration of the therapeutic potential of UAE’s medicinal flora. Although several plants in the UAE exhibit potential medicinal properties, this review focuses on *N. sativa* and *Z. lotus* due to their extensive pharmacological documentation, cultural relevance, and strong evidence supporting their antidiabetic and antimicrobial effects. Additionally, comprehensive reviews on such native plants are limited in the UAE, further justifying our focused selection.

### 2.2 Inclusion criteria

This review includes studies that focus on the antidiabetic and antimicrobial properties of medicinal plants native to or widely utilized in the UAE. Studies were selected based on the following criteria:

Scope of Research: Peer-reviewed articles, experimental studies (*In vitro* and *In vivo*), observational studies, ethnobotanical surveys, and reviews that provide pharmacological, phytochemical, or ethnopharmacological insights into medicinal plants.

Relevance: Research explicitly addressing the medicinal applications of UAE flora, particularly in managing diabetes, and combating bacterial infections.

Phytochemical Data: Studies providing detailed analysis of active bio-compounds, mechanisms of action, and their demonstrated therapeutic efficacy.

Temporal Range: Articles published within the last 20 years, ensuring contemporary data and relevance.

Language: Publications in English or those with reliable translations to maintain the accuracy of data interpretation.

The 20-year temporal range was selected to encompass recent pharmacological advancements while retaining historical context relevant to traditional practices. They are widely utilized in the UAE, which refers to plants frequently cited in ethnobotanical surveys, traditional medicine literature, and regional healthcare practices. While every effort was made to ensure comprehensive coverage, the review may have excluded relevant non-English publications lacking reliable translations, which is acknowledged as a potential limitation in capturing all available data.

### 2.3 Exclusion criteria

Studies were excluded if they did not align with the following parameters:

Geographic Focus: Research on plants that are not native to or widely adapted for traditional or modern medicinal use in the UAE.

Medicinal Relevance: Articles focusing solely on nutritional, agricultural, or ornamental aspects of plants without discussing their medicinal applications.

Scientific Credibility: Non-peer-reviewed publications, anecdotal reports, or sources with limited scientific rigor were omitted to ensure data reliability.

Scope of Data: Studies lacking sufficient pharmacological or phytochemical data, such as those not identifying bioactive compounds, mechanisms of action, or relevant therapeutic outcomes.

Health Focus: Research that does not specifically address antidiabetic, or antimicrobial was excluded to maintain the study’s focus on key health challenges.

This rigorous inclusion and exclusion process ensures that the review synthesizes high-quality, relevant data to advance the understanding of UAE MPs’ therapeutic potential (Figure 1).

### 2.4 PRISMA flow and article selection summary

**Figure 2:**
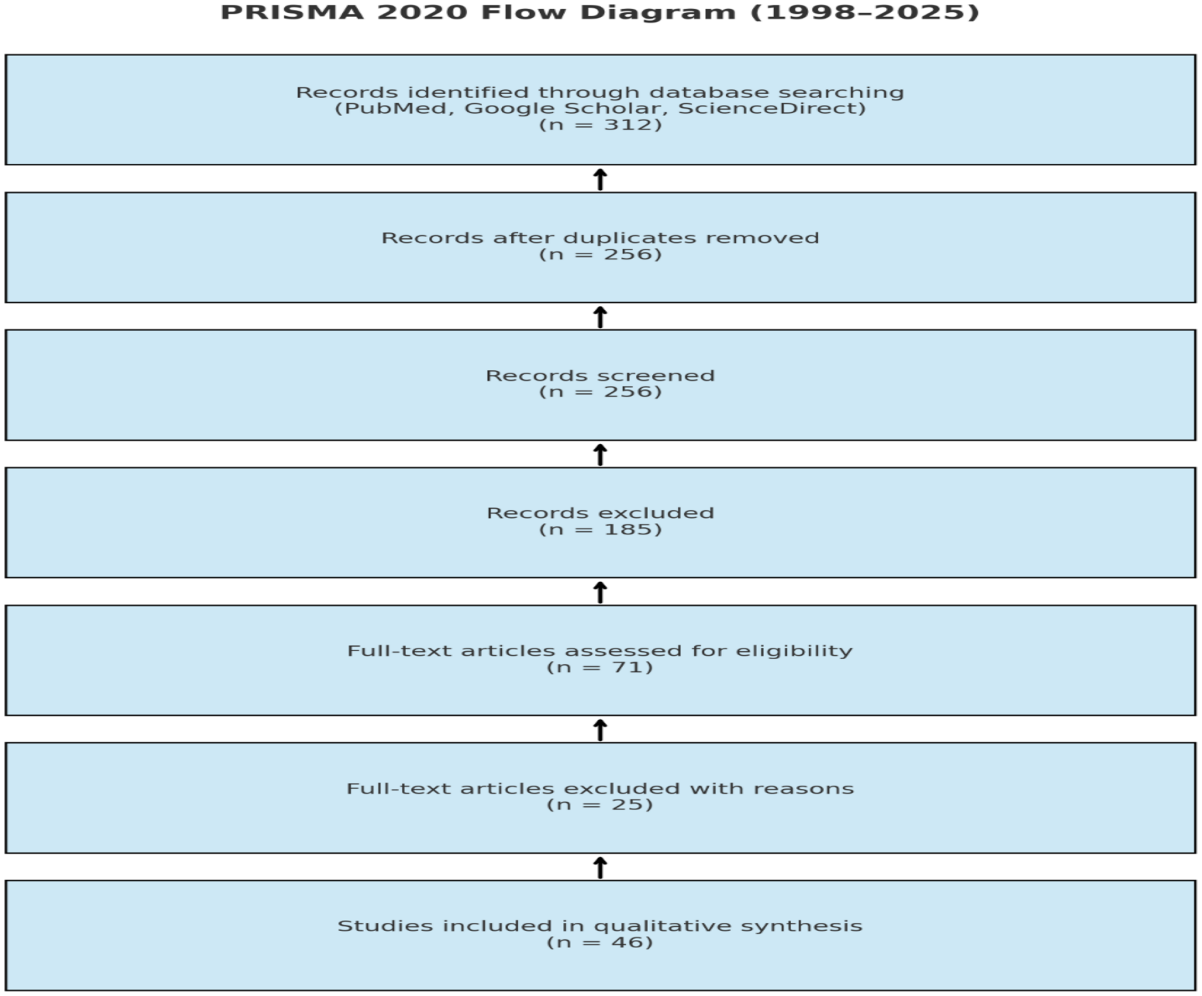
PRISMA Flow Diagram Illustrating the Literature Search and Selection Process.

Following the PRISMA 2020 (Preferred Reporting Items for Systematic Reviews and Meta-Analyses) guidelines, a systematic literature search was conducted across three major electronic databases: PubMed, Google Scholar, and ScienceDirect. The search targeted studies published between 1998 and 2025, focusing on the antidiabetic and antimicrobial properties of *N. sativa* and *Z. lotus*. A total of 312 records were initially retrieved. After the removal of duplicates, 256 articles remained for title and abstract screening. Of these, 185 articles were excluded for not meeting the inclusion criteria. Subsequently, 71 full-text articles were assessed for eligibility, with 25 articles excluded due to methodological limitations or insufficient relevance. Ultimately, 46 studies were deemed suitable and were included in the qualitative synthesis. The full selection process is depicted in the PRISMA flow diagram (Figure 2), ensuring transparency, reproducibility, and methodological rigor.

## 3 Medicinal plants in the UAE overview

### 3.1 Nigella sativa

*Nigella sativa,* commonly known as black seeds or black cumin, is an annual herb belonging to the genus Nigella in the family Ranunculaceae. Cultivated on private farms in the UAE, *N. sativa* has been widely recognized for its applications in traditional and contemporary medicine, as well as in culinary uses (25–27). Its seeds are rich in bioactive compounds, including thymoquinone, p-cymene, carvone, citronellol, nigellimine, and nigellidine. These compounds contribute to its significant pharmacological properties, including antioxidant, anti-inflammatory, antidiabetic, and hepatoprotective effects (28–32).

Pharmacological studies have demonstrated its potential in managing diabetes, hypertension, hypercholesterolemia, and antimicrobials. Antimicrobial investigations have demonstrated its efficacy against a broad spectrum of pathogens, attributed to the presence of thymoquinone and thymol. This includes significant activity against *S. aureus, E. coli,* and *P. aeruginosa*, in addition to its notable antifungal properties against *Candida albicans (C. albicans)* (33–36). Moreover, its antiviral effects, such as protection against murine cytomegalovirus, have been attributed to its ability to enhance macrophage function and interferon-gamma production (37). *N. sativa* also exhibits antidepressant and anticonvulsant properties, along with cytotoxic, anti-proliferative, and pro-apoptotic effects, supporting its potential use in cancer therapies (38–40).

Additionally, its hepatoprotective effects have been validated in experimental studies, where it showed protective effects against induced hepatotoxicity while improving serum lipid profiles (41). These findings affirm *N. sativa* as a valuable medicinal plant for preventive and therapeutic applications across a broad range of diseases (Table 1).

**Table 1:** Active compounds of *Nigella sativa* and *Ziziphus lotus.*

Scientific name	Common name	UAE flora	Active compound	Reference
*N. sativa*	(Black seed/black cumin)	Cultivated	Thymoquinone, P-cymene Quercetin Nigellimine, Nigellidine	(28, 30–32)
*Z. lotus*	Sedra	Native	Cyclopeptide alkaloids (lotusines), Dammarane saponins, Pentacyclic triterpenic compound, Sterols, Tocopherols, Flavonoids, Tannins, Anthocyanins, Coumarins, Saponins, Sterols, Deoxysugars, Mucilages	(27, 43, 44, 61)

*N. sativa*, *Nigella sativa*; *Z. lotus*, *Ziziphus lotus*.

### 3.2 Ziziphus lotus

*Ziziphus lotus,* deciduous shrub commonly known as “jujube” and “sidr” belongs to the genus Zizyphus of the angiosperm Rhamnaceae family and is a perennial MP native to the UAE, thriving on stony slopes and alluvial plains (27, 42–44). This medicinal fruit, which flowers from March to April, has garnered significant scientific interest due to its rich composition of bioactive secondary metabolites, mainly phenolic compounds in various plant parts (27, 44). Extensive research has revealed various biological properties of *Z. lotus* polar extracts, including antioxidant, antibacterial, anti-ulcerogenic, anti-inflammatory, analgesic, antidiabetic, and antispasmodic activities (43–47). While the less polar fractions of *Z. lotus* remain relatively unexplored, studies have identified several unique compounds such as cyclopeptide alkaloids (lotusines), dammarane saponins, and a pentacyclic triterpenic compound in different plant tissues. Additionally, sterols and tocopherols have been evaluated in *Z. lotus* seed oils and whole fruits, though some analyses lack quantification (44). The diverse phytochemical profile and potential therapeutic applications of *Z. lotus* underscore its importance as a subject of ongoing research in the field of natural products and MPs (Table 1).

### 3.3 Traditional use and Ethnopharmacological relevance in the UAE

The use of Complementary and Alternative Medicine (CAM) among patients with T2DM is notably high in the UAE, reflecting deep-rooted cultural practices and a growing inclination toward natural health solutions. A cross-sectional study involving T2DM patients from Dubai and Sharjah revealed that 39.3% had used CAM since diagnosis mainly folk foods and herbs often without physician guidance, and mostly influenced by family or social networks (48). *N. sativa*, one of the most widely recognized herbal medicines in the region, is acknowledged by community pharmacists in Dubai for its therapeutic potential despite regulatory and practice challenges (49). Likewise, *Z. lotus*, a native plant traditionally used to manage diabetes, infections, and skin conditions, has been shown to possess pharmacologically active compounds supporting these folk uses (27, 50). Recent comprehensive ethnopharmacological reviews have highlighted UAE-native plants such as *Z. lotus*, *N. sativa*, *Phoenix dactylifera*, *Trigonella foenum-graecum*, and *Withania somnifera* for their bioactive properties, showing relevance for addressing metabolic, cardiovascular, and antimicrobial conditions (51, 52). Additionally, a recent national study revealed strong public engagement with probiotics and prebiotics, especially among younger adults, reinforcing interest in plant-based functional products (53). Expanding the scope of plant-based medicine, modern technologies such as nano-drug delivery and 3D bioprinting are being explored to enhance the therapeutic delivery of natural compounds, including those used for conditions like alopecia underscoring the wider biomedical relevance of phytotherapy (54).

### 3.4 Geographical origin and phytochemical variability

The medicinal plants discussed in this review, particularly *N. sativa* and *Z. lotus*, are either native to the UAE or widely cultivated and used across the Middle East, Africa, and parts of Europe and Asia. *Z. lotus*, for example, is native to the arid and semi-arid regions of North Africa and the Mediterranean basin, including Algeria and Morocco, and is considered indigenous to the UAE (27). *N. sativa*, while widely cultivated in the UAE, is not native to the region but has a long history of traditional use across Asia, Eastern Europe, and North Africa (52, 55). Its seeds are extensively used in Middle Eastern and South Asian diets and ethnomedicine.

Geographical origin plays a significant role in the phytochemical profile of medicinal plants, influencing their bioactive potential and therapeutic efficacy. In the case of *N. sativa*, comparative studies have shown that seeds grown in different countries (India, Syria, Egypt, Poland) vary significantly in thymoquinone, tocotrienols, sterols, and terpene content, with some regions producing oil with higher antioxidant capacity and different antimicrobial potential (56). Similarly, the metabolic output of *Z. lotus* may be influenced by environmental conditions and microbial associations, including endophytic fungi, which differ by region and contribute to the antioxidant and antibacterial activity of the plant (14). These variations underscore the importance of location-specific studies and standardization, especially when formulating nutraceuticals or conducting comparative pharmacological research.

Thus, while the UAE context provides a unique environmental and cultural framework for the traditional use of these plants, the bioactive composition of *N. sativa* and *Z. lotus* can differ significantly depending on their country of origin, cultivation conditions, and ecological microbiota. Future studies should integrate chemical profiling with geographical data to ensure therapeutic consistency and efficacy across different formulations.

## 4 Overview of antidiabetic properties of medicinal plants in the UAE

### 4.1 Nigella sativa

*Nigella sativa* contains thymoquinone, a principal bioactive compound that has demonstrated significant antidiabetic properties through multiple mechanisms of action. The administration of thymoquinone results in a marked reduction of plasma glucose levels. This hypoglycemic effect is thought to be mediated through two primary pathways: firstly, thymoquinone appears to increase insulin levels, potentially by stimulating insulin secretion or improving insulin sensitivity, and secondly, it enhances the activities of various cytosolic and mitochondrial enzymes involved in glucose metabolism. These enzymatic modulations likely contribute to improved glucose utilization and cell energy production (30, 57, 58). Increasing insulin availability and optimizing cellular glucose metabolism underscore thymoquinone’s potential as a promising natural compound for diabetes management. Research evidence supports the antidiabetic potential of *N. sativa* and its primary bioactive compound, thymoquinone (32). An experimental animal study using adult female streptozotocin-induced diabetic Wistar rats demonstrated significant reductions in glucose levels, body weight, and insulin levels following the administration of 10 mg/kg of *N. sativa* extract (thymoquinone) (59). Furthermore, administering 2 mL/kg of *N. sativa* oil for 30 days resulted in significant improvements in fasting blood glucose, insulin, and lipid profiles in streptozotocin-induced diabetic male Wistar rats (60). Additionally, the synergistic effect of *N. sativa* with exercise regimens has been explored, with two human studies and two animal studies showing that combined administration of *N. sativa* and exercise produced more pronounced benefits in glycemic and lipidemic control compared to exercise alone (25). Additionally, *in vivo* and *in vitro* studies have consistently demonstrated the positive effects of *N. sativa* against diabetes (26). These findings underscore the potential of *N. sativa* and its constituents as promising natural interventions for diabetes management, warranting further investigation in clinical settings (Table 2).

**Table 2:** Antidiabetic properties of thymoquinone in *Nigella sativa.*

Study Focus	Findings	Methodology	Reference
Insulin levels and glucose metabolism	Thymoquinone increased insulin levels and enhanced the activities of enzymes involved in glucose metabolism.	*In vivo*	(30, 57, 58)
Reduction in glucose levels	Significant reductions in glucose levels, body weight, and insulin levels in streptozotocin-induced diabetic Wistar rats.	Experimental animal study	(59)
Combined effects of exercise	More pronounced benefits in glycemic and lipidemic control with combined administration of *N. sativa* and exercise compared to exercise alone.	Human and animal studies	(25)
General antidiabetic potential	*In vivo* and *In vitro* studies demonstrate positive effects against diabetes.	Review of multiple studies	(26)

*N. sativa, Nigella sativa.*

### 4.2 Ziziphus lotus

*Ziziphus lotus* is a rich source of bioactive compounds, including flavonoids, tannins, anthocyanins, coumarins, saponins, sterols, deoxysugars, and mucilages, contributing to its diverse pharmacological properties (42, 61). These phytochemicals, particularly its high polyphenol content, are known for their antioxidant, antimicrobial, and immunomodulatory effects (62). Research has demonstrated the significant anti-diabetic potential of *Z. lotus* extracts. *In vivo* studies showed that an aqueous extract of *Z. lotus* fruit (300 mg/kg) effectively controlled blood glucose levels in diabetic hamsters, with efficacy comparable to metformin (45). Furthermore, *In vitro* investigations revealed potent inhibitory effects of *Z. lotus* leaf and fruit extracts on *α*-amylase (IC50 = 20.40–31.91 μg/mL) and α-glycosidase (IC50 = 8.66–27.95 μg/mL), surpassing the efficacy of acarbose (45). The traditional use of *Z. lotus* as an anti-diabetic agent is supported by ethnobotanical surveys in Morocco, where it is among the plants used by traditional healers to treat diabetes (63). Environmental factors have been shown to significantly influence the plant’s secondary metabolites, affecting its bioactivities, which further supports the need for location-specific cultivation to optimize its medicinal value (64). Additionally, metabolomic studies have provided insights into the metabolic diversity and potent *in-vitro* antidiabetic potential of *Z. lotus*, identifying several bioactive metabolites that could contribute to its therapeutic effects (65). Moreover, the plant has shown significant effects on human T-cell proliferation, indicating its potential in modulating immune responses. The fruit pulp and seeds, in particular, were found to have higher concentrations of vitamins and fatty acids, which contributed to its notable antioxidant capacity and beneficial immunosuppressive effects, particularly in reducing excessive immune responses and inflammation (66). These findings underscore *Z. lotus’s* potential for nutraceutical/pharmaceutical applications in food formulations and pharmaceutical development, warranting further investigation in clinical settings (Table 3).

**Table 3:** Evaluation of *Ziziphus lotus* extracts for antidiabetic efficacy and biochemical properties.

Study Focus	Findings	Methodology	Reference
Control of blood glucose	Aqueous extract of *Z. lotus* fruit effectively controlled blood glucose levels in diabetic hamsters, with efficacy comparable to metformin.	*In vivo* study on diabetic hamsters	(45)
Enzyme inhibition	Potent inhibitory effects on *α*-amylase and *α*-glycosidase; extracts surpassed the efficacy of acarbose.	*In vitro* investigations	(45)
Ethnobotanical relevance	Used by traditional healers in Morocco to treat diabetes.	Ethnobotanical survey	(63)
Metabolic diversity and antidiabetic potential	Metabolomic studies identified bioactive metabolites contributing to therapeutic effects.	Metabolomic studies	(65)
Antioxidant capacity and immunosuppressive effects	Higher concentrations of vitamins and fatty acids in the fruit pulp and seeds contributed to notable antioxidant and immunosuppressive effects.	Biochemical analysis	(66)

*Z. lotus*, *Ziziphus lotus*.

## 5 Overview of antibacterial properties of medicinal plants in the UAE

### 5.1 Nigella sativa

*Nigella sativa* has garnered substantial attention for its extensive antimicrobial properties, which have been rigorously documented in various studies. Shafodino et al. (67) highlighted the remarkable antimicrobial potential of *N. sativa* seed extracts, employing advanced techniques such as Gas Chromatography–Mass Spectrometry (GC–MS) and Fourier-Transform Infrared Spectroscopy (FTIR) to identify active compounds with significant activity against pathogens such as *E. coli* and *S. aureus* (67). In parallel, Hossain et al. (68) emphasized the role of key bioactive constituents, including thymoquinone, in combatting microbial resistance by inducing oxidative stress and triggering apoptosis in microbial cells (68).

Similarly, Al-Ameedy and Omran (69) demonstrated the potent antibacterial and antifungal activities of hexane and ethanol seed extracts, underscoring their potential in addressing infectious diseases (69). The work of Abbas et al. (28) further substantiates the plant’s therapeutic promise, showing that phytochemicals like quercetin in *N. sativa* achieve significant minimum inhibitory concentrations (MIC) against multidrug-resistant strains, including *methicillin-resistant Staphylococcus aureus* (MRSA), thus highlighting the plant’s efficacy in addressing antibiotic resistance (28). Studies by Ashraf et al. (70) and Tiji et al. (71) lend additional credence to these findings, linking the antimicrobial effects of *N. sativa* to its diverse phytochemical composition and efficacy against a broad spectrum of bacterial and fungal species (70, 71).

Further expanding the evidence base, Kocoglu et al. (72) demonstrated the utility of *N. sativa* oil in effectively combating bacteria implicated in otitis media and external (72). Likewise, Kolayli et al. (73) revealed the unique antimicrobial properties of *N. sativa* honey, particularly against *Listeria monocytogenes* (*L. monocytogenes*) and *S. aureus*, further broadening its therapeutic applications (73). Elmowalid et al. (74) illustrated the immunomodulatory properties of *N. sativa*, showcasing its potential to enhance immune response and improve growth performance in rabbits exposed to MRSA infections (74).

Of particular interest, Bhatti et al. (31) explored the role of phytochemicals such as thymoquinone and p-cymene in disrupting microbial cell membranes and inhibiting cellular division, highlighting the plant’s environmentally friendly approach to combating microbial infections (31). In a similar vein, Bhavikatti et al. (75) elucidated the significant antimicrobial activity of *N. sativa* essential oil (NSEO) against oral pathogens. Their study revealed that NSEO inhibits protein denaturation and stabilizes cell membranes, demonstrating antimicrobial efficacy comparable to chlorhexidine against *E. coli* and *S. aureus*, with enhanced effects on *Lactobacillus acidophilus* and *C. albicans*. These findings underscore NSEO’s potential as a therapeutic agent for managing oral infections, particularly those resistant to conventional treatments. Collectively, these studies underscore the immense potential of *N. sativa* as a natural antimicrobial agent, advocating for further exploration into its broad-spectrum therapeutic applications. The cumulative evidence not only reinforces its role in addressing the global challenge of antibiotic resistance but also positions it as a cornerstone in the development of sustainable antimicrobial strategies (75) (Table 4; Figure 1).

**Table 4:** Antimicrobial research on *Nigella sativa* targeting various microorganisms.

Scientific name	Title	Trial type	Model or method	Used part or form	Target microorganisms	Therapeutic effect	Reference
*Nigella sativa*	Potent antimicrobial activity of different extracts of *N. sativa* seeds	*In vitro*	GC–MS, FTIR	Seeds extracts	*E. coli, S. aureus*	Antimicrobial	(67)
	Bioactive compounds of *N. sativa* and their antimicrobial resistance mechanisms	*In vitro*	Biochemical assays	Bioactive compounds	General microbial resistance	Antimicrobial	(68)
	Antifungal and antibacterial activities of hexane and ethanol extracts of *N. sativa* seeds	*In vitro*	Disc diffusion	Hexane, ethanol extracts	Various fungi and bacteria	Antifungal, Antibacterial	(69)
	Antimicrobial efficacy of *N. sativa* compounds against MRSA	*In vitro*	MIC assays	Compounds	MRSA	Antimicrobial	(28)
	Antimicrobial efficacy of *N. sativa* against bacterial and fungal species	*In vitro*	Agar-well diffusion	Not specified	Various bacterial and fungal species	Antimicrobial	(70)
	Link between phytochemical composition and antimicrobial activity of *N. sativa*	Review	Review of studies	Not specified	General microbial species	Antimicrobial	(71)
	Effectiveness of *N. sativa* oil against common bacteria in otitis media and externa	*In vitro*	Disc diffusion	Oil	Bacteria in otitis media and externa	Antibacterial	(72)
	Antimicrobial properties of *N. sativa* honey against *L. monocytogenes and S. aureus*	*In vitro*	Agar-well diffusion	Honey	*L. monocytogenes, S. aureus*	Antimicrobial	(73)
	Modulating immune responses and enhancing growth performance in rabbits with *N. sativa*	*In vivo*	Animal model	Not specified	MRSA	Immunomodulatory	(74)
	Phytochemicals of *N. sativa* disrupting microbial cell membranes	*In vitro*	Membrane assays	Phytochemicals	General bacteria	Antimicrobial	(31)
	Antimicrobial activity of *N. sativa* essential oil against oral pathogens	*In vitro*	Protein denaturation assay	Essential oil	*E. coli, S. aureus, L. acidophilus, C. albicans*	Antimicrobial	(75)

*N. sativa*, *Nigella sativa*; *E. coli*, *Escherichia coli*; *S. aureus*, *Staphylococcus aureus*; *L. acidophilus*, *Lactobacillus acidophilus*; *C. albicans*, *Candida albicans*; MRSA, methicillin-resistant *S. aureus*; MIC, minimum inhibitory concentrations; *L. monocytogenes*, *Listeria monocytogenes*.

### 5.2 Ziziphus lotus

*Ziziphus lotus* has been shown to contain a diverse array of bioactive compounds, including flavonoids, tannins, anthocyanins, coumarins, saponins, sterols, deoxysugars, and mucilages (43, 45). These phytochemicals contribute to the plant’s significant antimicrobial properties, which have been demonstrated across multiple studies using various extracts and methodologies (42, 61). Notably, acetonic extracts of *Z. lotus* leaves exhibited potent antibacterial activity against several strains, including Notably, acetonic extracts of *Z. lotus* leaves exhibited potent antibacterial activity against several strains, including MRSA.

**Figure 3:**
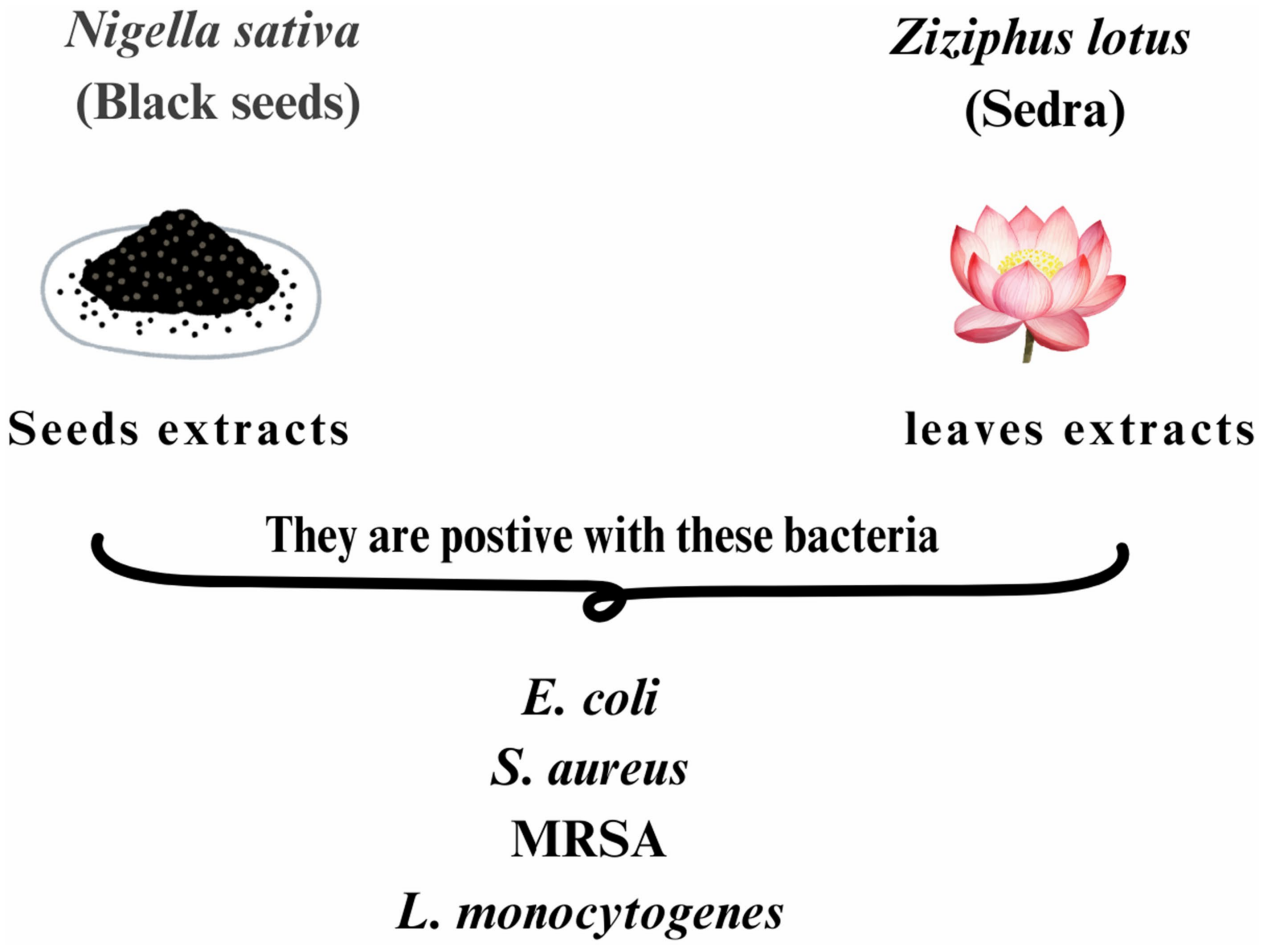
Antibacterial Activity of *Nigella sativa* and *Ziziphus lotus* extracts against *Escherichia coli* (*E. coli*), *Staphylococcus aureus* (*S. aureus*), *methicillin-resistant S. aureus* (MRSA), and *Listeria monocytogenes* (*L. monocytogenes*).

Methanolic and ethanolic extracts from different plant parts also showed broad-spectrum antimicrobial effects against both gram-positive and gram-negative bacteria, including *E. coli, P. aeruginosa,* and *L. monocytogenes,* with MIC values varying widely, from as low as 3.2 μg/mL to 200 mg/mL depending on the bacterial strain and extract type (47, 76). Additionally, the antimicrobial efficacy of *Z. lotus* extracts has been consistently demonstrated using various methods, including disc diffusion, agar-well diffusion, and microdilution techniques (46). These findings collectively underscore the potential of *Z. lotus* as a rich source of bioactive compounds. Its antimicrobial efficacy is notably influenced by environmental factors such as climate, soil conditions, and geographic origin, highlighting a critical area for future research to optimize medicinal value through location-specific cultivation strategies (42, 64) (Table 5; Figure 3).

**Table 5:** Studies on the antimicrobial effects of *Ziziphus lotus.*

Scientific name	Title	Trial type	Model or method	Used part or form	Target microorganisms	Therapeutic effect	Reference
*Ziziphus lotus*	Bioactive compounds contributing to antimicrobial properties	Review	Review of studies	Various parts	General bacterial and fungal species	Antimicrobial	(45)
	Broad-spectrum antimicrobial effects of *Z. lotus* extracts	*In vitro*	Disc diffusion	Extracts	*E. coli, P. aeruginosa, L. monocytogenes*	Antimicrobial	(43)
	Variability in MIC values of *Z. lotus* extracts against bacteria	*In vitro*	MIC assays	Extracts	*E. coli, P. aeruginosa, L. monocytogenes*	Antimicrobial	(47)
	Antimicrobial efficacy of *Z. lotus* extracts demonstrated by various methods	*In vitro*	Various methods	Extracts	Various bacterial and fungal species	Antimicrobial	(76)
	Antioxidant and antimicrobial activities of *Z. lotus*	*In vitro*	Antioxidant assays	Not specified	General bacterial and fungal species	Antimicrobial, Antioxidant	(46)
	Environment effect on antimicrobial activity of *Z. lotus*	Observational	Environmental study	Not specified	General bacterial and fungal species	Antimicrobial	(64)
	Consistent demonstration of antimicrobial efficacy of *Z. lotus* extracts	Review	Review of studies	Extracts	General bacterial and fungal species	Antimicrobial	(42)

*N. sativa*, *Nigella sativa*; *Z. lotus*, *Ziziphus lotus*; *E. coli*, *Escherichia coli*; *S. aureus*, *Staphylococcus aureus*; *P. aeruginosa*, *Pseudomonas aeruginosa*; *L. monocytogenes*, *Listeria monocytogenes*.

## 6 Computational investigations

Recent computational investigations have significantly contributed to understanding the therapeutic potential of bioactive compounds from *N. sativa* and *Z. lotus*. For example, a network pharmacology and molecular docking study identified *N. sativa* compounds such as kaempferol and catechin as multi-target agents for diabetes and obesity management. These compounds exhibited strong binding affinities to AKT1 and other diabetes-related targets like IL6 and EGFR (77). In another study, components of *N. sativa* essential oil, such as *α*-phellandrene and thymol, showed notable inhibition of α-amylase and hemoglobin glycation, supported by in silico docking analyses (78).

A recent virtual screening of *N. sativa* phytoconstituents against the angiotensin II type 1 receptor (AT1R) identified *β*-amyrin and taraxerol as potential candidates for diabetic-hypertensive therapy. Molecular dynamics simulations confirmed their stable binding and favorable pharmacokinetic profiles (79). Additionally, computational studies have explored the antiviral potential of dithymoquinone (DTQ) from *N. sativa* against SARS-CoV-2, with strong docking affinity to the ACE2 binding site, supported by MM-PBSA and molecular dynamics simulations (80, 81).

From an antimicrobial perspective, *N. sativa* extracts have been analyzed in silico for their interactions with bacterial quorum-sensing proteins such as LasR and PqsR. Thymol and thymoquinone displayed strong binding affinities, correlating with observed *in vitro* antimicrobial synergy (36). Another study combined in vitro and in silico evaluations of silver nanoparticles synthesized from *N. sativa* to demonstrate effective inhibition of CTX-M-15—a beta-lactamase enzyme linked to antibiotic resistance (82).

Similarly, *Z. lotus* has been computationally evaluated for its antidiabetic and antimicrobial potential. A metabolomics-based study identified bioactive metabolites such as caffeic acid, betulinic acid, quercetrin, and jujubogenin from *Z. lotus* and *Zizphus spina-christi*, which showed inhibitory effects against *α*-amylase and α-glucosidase in vitro. These findings were supported by multivariate analysis and molecular modeling (65). In a related study, betulin and betulinic acid isolated from *Ziziphus spina-christi* showed strong docking interactions with microbial targets, aligning with their antimicrobial activity (83).

Furthermore, the phenolic-rich stem bark extract of *Z. lotus* showed tyrosinase-inhibitory activity and potent antioxidant effects. Molecular docking confirmed the strong binding of these phytoconstituents to oxidative stress and skin-aging targets (84).

Collectively, these computational studies underscore the multifaceted therapeutic potential of phytoconstituents from *N. sativa* and *Z. lotus*, and highlight their relevance in drug discovery for metabolic, microbial, and viral diseases.

## 7 Molecular mechanisms of bioactive compounds: *in vitro, in vivo*, and clinical evidence

The therapeutic efficacy of *N. sativa* and *Z. lotus* is supported by mounting evidence spanning *in vitro* assays, animal models, and limited clinical trials. At the molecular level, the secondary metabolites of *N. sativa*, particularly thymoquinone, exhibit multifaceted biological activities. *In vitro* studies have demonstrated thymoquinone’s ability to inhibit *α*-amylase and α-glucosidase, reduce hemoglobin glycation, and scavenge free radicals (78). These effects correlate with its observed capacity to modulate oxidative stress pathways and improve insulin sensitivity in cell-based assays (85).

*In vivo*, thymoquinone improves glucose tolerance, reduces hepatic gluconeogenesis, and enhances antioxidant enzyme activity (SOD, CAT, GPx) in diabetic animal models (57, 85). It also upregulates insulin-like growth factor-1 and suppresses inflammatory mediators such as cyclooxygenase-2 (COX-2), supporting its anti-inflammatory and insulinotropic actions (85). Moreover, in silico studies have confirmed thymoquinone’s strong binding affinity to microbial virulence regulators (e.g., LasR, PqsR) and human diabetes-related targets (e.g., AKT1), supporting its dual application as an antidiabetic and antimicrobial agent (36).

Clinically, *N. sativa* supplementation has been shown to reduce fasting blood glucose, HbA1c, and improve lipid profiles in several small-scale trials, though more robust and inclusive studies are needed to validate its pharmacokinetics, therapeutic dose range, and long-term safety (85, 86).

Regarding *Z. lotus*, its bioactivity has been attributed to a diverse array of polyphenols, flavonoids, and triterpenoids. *In vitro*, extracts have demonstrated significant *α*-glucosidase and α-amylase inhibition, along with antioxidant and anti-inflammatory effects (45, 87). Polyphenols such as rutin, hyperin, and isoquercitrin are known to regulate lipid metabolism, inhibit NF-κB signaling, and improve insulin resistance (88) Betulinic acid and related triterpenoids from *Z. lotus* also show promising *in vitro* antimicrobial and cytotoxic effects (83).

*In vivo* studies have confirmed the antihyperglycemic and hepatoprotective effects of *Z. lotus* extracts in diabetic and hyperlipidemic animal models, with upregulation of antioxidant enzymes and downregulation of TNF-α and IL-1β (88) Additionally, ethanolic extracts of *Z. lotus* showed potent antimicrobial activity against *S. aureus*, *E. coli*, and *C. albicans* in comparative MIC-based assays, validating its traditional use against infectious diseases (87, 89).

While clinical trials on *Z. lotus* remain limited, the plant’s ethnomedicinal record, chemical diversity, and consistent *In vitro/In vivo* data suggest its potential as a therapeutic candidate. Ongoing metabolomic and computational studies continue to validate its mechanisms, particularly in metabolic syndrome and microbial infections (65).

Collectively, the integration of *In vitro, In vivo,* and early-stage clinical findings supports the pharmacological versatility of both plants. These data reinforce the need for translational research and well-designed human trials to optimize bioavailability, safety, and therapeutic efficacy. A summary of the molecular mechanisms across *In vitro, In vivo*, and clinical trials is presented in Tables 6, 7.

**Table 6:** Molecular mechanisms of *Nigella sativa* and *Ziziphus lotus* in diabetes management.

Plant	Experimental level	Mechanism of action/findings	Key bioactive compounds	Reference
*N. sativa*	*In vitro*	Inhibits α-amylase and hemoglobin glycation; strong antioxidant and antiradical activity	Thymoquinone, α-phellandrene, thymol	(78)
*N. sativa*	*In vivo*	Enhances insulin secretion; reduces gluconeogenesis; upregulates IGF-1; reduces oxidative stress and inflammation	Thymoquinone	(85)
*N. sativa*	Clinical trials	Reduces fasting glucose and HbA1c; improves lipid profile	Thymoquinone-rich extracts	(86)
*Z. lotus*	*In vitro*	Inhibits α-amylase and α-glucosidase; antioxidant and anti-inflammatory activity	Rutin, quercetin, triterpenoids	(45, 90)
*Z. lotus*	*In vivo*	Lowers blood glucose and lipids; modulates TNF-α, IL-1β, TGF-β1	Betulinic acid, polyphenols	(90)

*N. sativa*, *Nigella sativa*; *Z. lotus, Ziziphus lotus*.

**Table 7:** Molecular mechanisms of *Nigella sativa* and *Ziziphus lotus* in antimicrobial activity.

Plant	Experimental level	Mechanism of action/findings	Key bioactive compounds	Reference
*N. sativa*	In silico	Strong binding to AKT1, LasR, PqsR; relevant to diabetes and microbial inhibition	Thymoquinone, thymol	(36)
*Z. lotus*	In silico	Docking confirms binding to microbial and metabolic targets	Betulin, triterpenes	(83)
*Z. lotus*	Antimicrobial assays	Strong activity against *S. aureus*, *E. coli*, *C. albicans* (MIC ≤ 0.012 mg/mL)	Ethanol extract (fruits, flowers)	(87, 89)

*N. sativa*, *Nigella sativa*; *Z. lotus*, *Ziziphus lotus*; *E. coli*, *Escherichia coli*; *S. aureus*, *Staphylococcus aureus*; *C. albicans*, *Candida albicans*.

## 8 Discussion and conclusion

This review highlights robust pharmacological evidence supporting the therapeutic applications of *N. sativa* and *Z. lotus*, two UAE-native medicinal plants. Notably, thymoquinone from *N. sativa* has demonstrated a glucose-lowering effect comparable to or exceeding metformin in animal models, with improvements in fasting glucose and lipid profiles. *Z. lotus* extracts showed potent inhibition of *α*-amylase and α-glucosidase, surpassing acarbose in *in vitro* assays. Both plants also exhibited broad-spectrum antimicrobial activity against multidrug-resistant pathogens such as MRSA, *E. coli*, and *P. aeruginosa*, attributed to their rich phenolic and flavonoid content.

Comparative evaluation suggests that *N. sativa* may possess a higher overall therapeutic potential due to its well-documented antidiabetic, antimicrobial, antioxidant, and anti-inflammatory properties. Thymoquinone’s mechanisms include activation of AMPK, enhancement of insulin sensitivity, and modulation of oxidative and inflammatory pathways. Meanwhile, *Z. lotus* flavonoids and saponins demonstrate strong enzymatic inhibition and antimicrobial properties, though clinical and mechanistic evidence remains less extensive.

Both plants show targeted efficacy against T2DM, supported by streptozotocin-induced models and enzyme inhibition data. Their antimicrobial activity spans critical pathogens including MRSA, *E. coli*, *P. aeruginosa*, and *L. monocytogenes*, supporting their relevance in managing diabetes-associated infections and antimicrobial resistance.

Recent molecular studies highlight the role of *N. sativa* compounds in regulating glucose uptake, oxidative stress (via SOD, CAT, GPx), and bacterial virulence pathways (e.g., quorum sensing, biofilm formation). In *Z. lotus*, bioactive constituents downregulate pro-inflammatory cytokines and modulate MAPK and PI3K/Akt signaling, further supporting its dual therapeutic role.

**Figure 4:**
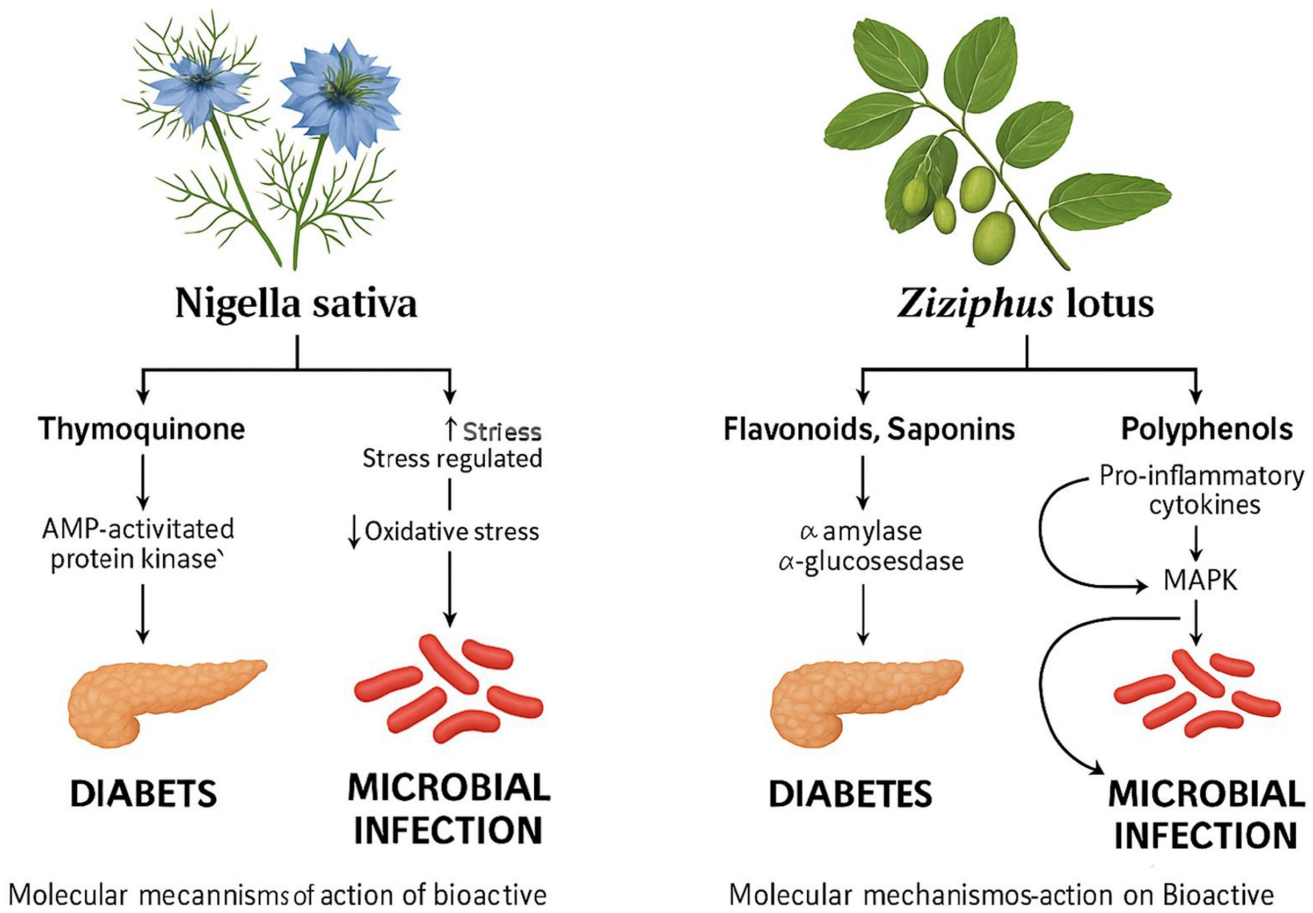
Molecular pathways illustrating the antidiabetic and antimicrobial effects of thymoquinone derived from *Ziziphus lotus* and *Nigella sativa*.

Altogether, these findings reinforce the need for well-controlled clinical trials to confirm efficacy and safety in human populations. Variability in phytochemical content driven by environmental conditions also underscores the importance of standardized cultivation and extraction protocols. Bridging traditional herbal knowledge with scientific validation, these plants hold strong potential for integration into sustainable, evidence-based healthcare strategies (Figure 4).

This diagram highlights how thymoquinone activates AMP-activated protein kinase (AMPK), enhances insulin sensitivity, reduces oxidative stress and inflammation in the management of T2DM. Additionally, it shows thymoquinone’s role in microbial inhibition through membrane disruption, efflux pump inhibition, and suppression of quorum sensing, leading to reduced biofilm formation and virulence gene expression.

## 9 Future perspectives

Looking ahead, research on *N. sativa* and *Z. lotus* should prioritize clinical validation, particularly given their demonstrated antidiabetic and antimicrobial efficacy in preclinical models. *N. sativa*’s thymoquinone and *Z. lotus* extracts have shown results that exceed conventional treatments like metformin and acarbose in experimental settings, justifying human trials to confirm therapeutic relevance and optimize dosage. Environmental factors influencing phytochemical composition should be explored to develop cultivation strategies that maximize medicinal potency. Furthermore, efforts to establish standardized extraction protocols and regulatory frameworks will be essential for integrating these native plants into mainstream healthcare. Finally, increased public and professional awareness of their therapeutic benefits can support policy development and sustainable use.
