# Supplementary material for: Therapeutic efficacy of Nigella sativa and Ziziphus lotus: sustainable strategies for diabetes, antimicrobial resistance, and health treatment
Source: Front Nutr. 2025 Sep 3;12:1592423. doi: 10.3389/fnut.2025.1592423 (PMC12442494; doi:10.3389/fnut.2025.1592423)
Supplement: Supplementary file 1 [file Supplementary_file_1.docx]

Supplementary Material

## Supplementary Figures


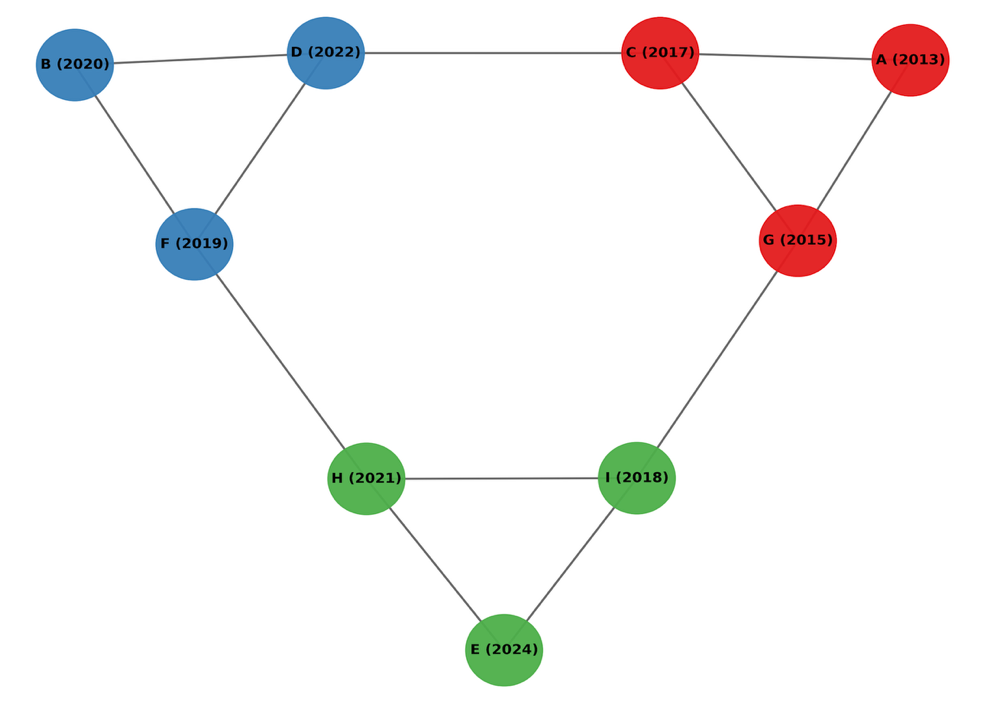


**Supplementary Figure 1.** Bibliographic Coupling Network of Reviewed Studies on *Nigella sativa* and *Ziziphus lotus*: Temporal Clustering and Citation Overlap.

**Footnote:**

This network visualizes bibliographic coupling among the reviewed articles related to the pharmacological and antimicrobial properties of *Nigella sativa* and *Ziziphus lotus*.
Each node represents a study and is labeled with a letter and publication year for clarity: Red cluster: Older foundational studies: A (2013), C (2017), G (2015), Blue cluster: Intermediate studies: B (2020), D (2022), F (2019), Green cluster: Recent studies: E (2024), H (2021), I (2018), Lines between nodes indicate shared citations, suggesting methodological or thematic similarities. The triangular spatial layout reflects temporal progression and thematic convergence among studies from different publication periods.
